# Development and Characterization of a Rabbit Model of Compromised Maxillofacial Wound Healing

**DOI:** 10.1089/ten.tec.2018.0361

**Published:** 2019-03-18

**Authors:** Stacey L. Piotrowski, Lindsay Wilson, Neeraja Dharmaraj, Amani Hamze, Ashley Clark, Ramesh Tailor, Lori R. Hill, Stephen Lai, F. Kurtis Kasper, Simon Young

**Affiliations:** ^1^ Department of Oral and Maxillofacial Surgery, School of Dentistry, The University of Texas Health Science Center, Houston, Texas.; ^2^ Center for Laboratory Animal Medicine and Care, The University of Texas Health Science Center, Houston, Texas.; ^3^ Department of Veterinary Medicine and Surgery, The University of Texas MD Anderson Cancer Center, Houston, Texas.; ^4^ Department of Diagnostic and Biomedical Sciences, School of Dentistry, The University of Texas Health Science Center, Houston, Texas.; ^5^ Department of Radiation Physics, The University of Texas MD Anderson Cancer Center, Houston, Texas; ^6^ Division of Surgery, Department of Head and Neck Surgery, The University of Texas MD Anderson Cancer Center, Houston, Texas.; ^7^ Department of Orthodontics, School of Dentistry, Graduate School of Biomedical Sciences, The University of Texas Health Science Center, Houston, Texas.

**Keywords:** irradiation model, bone tissue engineering

## Abstract

***Background:*** Tissue engineering technologies aiming to enhance maxillofacial wound healing are often tested *in vivo* in preclinical models that do not necessarily reflect the complexity of the clinical need. The aim of this study was to develop a rabbit model of compromised craniofacial wound healing that more accurately mimics clinical scenarios.

***Materials and Methods:*** An experimental group of rabbits received fractionated radiation of the mandible totaling 36 Gy. Four weeks after irradiation, both the experimental group and control group (*n* = 10/group) underwent a surgical procedure creating a critical size defect in the mandibular bone. Four weeks after surgery, tissue healing was assessed using microcomputed tomography (*μ*CT), maximum intensity projection (MIP) scoring, and histopathology.

***Results:*** *μ*CT analysis and MIP scoring showed decreased mineralized tissue in the defect area of irradiated animals compared to the control group. Histopathology showed necrosis in the experimental group.

***Conclusions:*** Irradiated animals showed significantly compromised wound healing compared to controls. This preclinical model presents a clinically relevant environment for the investigation of novel wound healing technologies in a compromised critical size bone defect.

**Impact Statement:**

Maxillofacial defects often present the clinical challenge of a compromised wound bed. Preclinical evaluation of tissue engineering techniques developed to facilitate healing and reconstruction typically involves animal models with ideal wound beds. The healthy wound bed scenario does not fully mimic the complex clinical environment in patients, which can lead to technology failure when translating from preclinical *in vivo* research to clinical use. The reported preclinical animal model of compromised wound healing enables investigation of tissue engineering technologies in a more clinically relevant scenario, potentially fostering translation of promising results in preclinical research to patients.

## Introduction

Injuries to the maxillofacial skeleton encompass a wide variety of ailments, from facial fractures to locally aggressive neoplasms such as ameloblastomas. Oral and maxillofacial reconstruction of tissue defects associated with these injuries is particularly challenging, due to a variety of factors, including repetitive motion to the affected area and possible exposure to the external environment in the mouth or sinuses.^1^ In addition, maxillofacial defects can often be associated with compromised wound beds, resulting in additional treatment difficulties due to factors such as decreased vascularization, wound contamination, or treatments such as radiation or chemotherapy.^2,3^ Failure of traditional treatment modalities such as tissue grafts and flaps in these compromised wound environments^4,5^ has prompted the continued investigation of tissue engineering technologies in hopes for improved wound healing therapies.^1^

Tissue engineering techniques seek to improve regeneration of damaged tissues through the combination of scaffolds, cells, and growth factors.^6,7^ A wide variety of scaffolds and tissue engineering technologies have been utilized for dentistry and maxillofacial surgery, including applications for periodontal tissue and bone regeneration.^4,8^ However, before clinical use of tissue engineering technologies in patients, *in vivo* testing in animal models is required to demonstrate potential translational success.^1^

Preclinical animal models for testing bone tissue engineering constructs include the creation of a critical size defect (CSD), a defect that will not heal during the natural lifetime of the animal.^9^ A wide variety of CSD models exist, in species ranging from rodents to dogs and goats and a multitude of bone types, including long bones and the calvarium.^10–12^ More specifically, tissue engineering technologies have been investigated in a CSD model in the rabbit mandible to mimic the oral and maxillofacial healing environment.^13,14^ However, these models typically involve the testing of tissue engineering technologies in a healthy wound bed that does not accurately mimic the previously described compromised oral wound environments seen clinically. While some animal models of compromised oral wound healing exist,^15,16^ lack of a CSD or possible challenges to reproducibility and consistency limit their utility in preclinical investigation of oral and maxillofacial tissue engineering technologies.

The aim of this study was to develop a preclinical model of compromised maxillofacial wound healing, which could be used for future evaluation of tissue engineering techniques. A previously described rabbit alveolar bone CSD model^17^ was combined with radiation, driven by the hypothesis that radiation would compromise the tissue healing in the model.

## Materials and Methods

### Animals

Twenty skeletally mature male New Zealand White rabbits, at least 6 months old and weighing 3.0–4.0 kg, were acquired from Charles River Laboratories (Wilmington, MA). Experimental manipulations were approved by the Institutional Animal Care and Use Committee of The University of Texas MD Anderson Cancer Center and the Animal Welfare Committee of The University of Texas Health Science Center. Animals were housed at an AAALAC-accredited facility in accordance with USDA regulations and the Guide for the Care and Use of Laboratory Animals by the United States National Research Council. Rabbits were allowed to acclimate for 1 week before experimental manipulations. Rabbits were randomly assigned to a nonirradiated control group or an irradiated experimental group (*n* = 10 in each group).

Upon arrival, the rabbits were fed standard pelleted feed (Purina LabDiet 5321, St. Louis, MO) and a variety of vegetables. In an effort to avoid mandibular fracture postsurgery, rabbits were transitioned to a softened diet consisting of ground pellets mixed with pureed fruits or vegetables, along with finely shredded vegetables.^18,19^ No clinical concerns were noted during the course of the study, and no animals were euthanized before the experimental endpoints.

### Irradiation

**FIG. 1.**
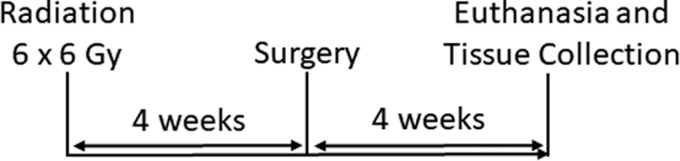
Study timeline.

Rabbits in the experimental group underwent radiation sessions while anesthetized with isoflurane (IsoThesia; Henry Schein Animal Health, Dublin, OH). Rabbits received 36 Gy of radiation, with a fractionation scheme of 6 × 6 Gy, targeted to the left mandible (Fig. 1). Radiation was administered thrice a week (Monday, Wednesday, Friday) for 2 weeks. Anesthetized rabbits were monitored through a video camera and computer feed viewed outside the irradiator room.

**FIG. 2.**
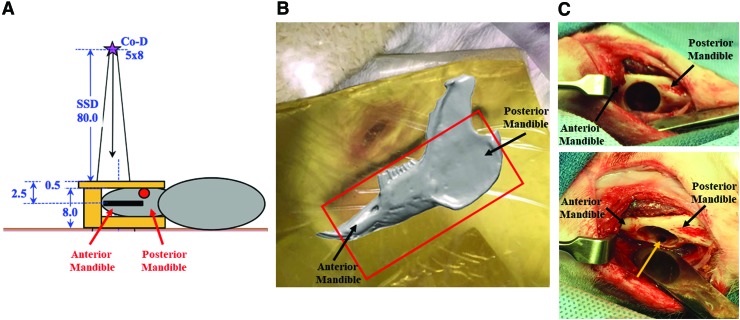
Protocol for mandibular radiation and surgical defect. **(A)** Schematic diagram of radiation positioning and distances to determine relevant measurements (in cm). *Purple star* is the cobalt radiation source (Co-D), with a radiation field size of 8 × 5 cm. SSD was 80 cm, with the mandible ∼2.5 cm below the top *surface*. **(B)** Photograph of radiation field with rabbit positioned in right lateral recumbency, covered with Superflab material and loosely packed dry rice as buildup materials. *Red outline* approximates the radiation field. **(C)** Intraoperative photographs of mandibular CSD. Rabbit is in dorsal recumbency. *Top panel*: Full thickness mandibular bone defect created with 10 mm trephine bur. *Bottom panel*: Intraoral communication (*yellow arrow*) created through overlying tooth crown with 1 mm cross cut bur. CSD, critical size defect; SSD, source-to-skin distance. Color images are available online.

Irradiations were performed using 2.2 MV gamma rays from a Cobalt-60 radiation-therapy machine (Co-D). Each rabbit was in the right lateral position with the head placed at an ∼45-degree angle in an attempt to spare other tissues. Radiation geometry was calculated using a radiation field size of 8 × 5 cm focused on the left mandible and nominal source-to-skin distance of 80 cm (Fig. 2A). To achieve a reproducible and uniform dose, air spaces surrounding the jaw were filled with “tissue-equivalent” materials to create a flat surface (Fig. 2B). The “tissue-equivalent” materials were composed of thin sealable bags filled with loosely packed dry rice and pieces of Superflab (Radiation Products Design, Inc., Albertville, MN), a synthetic gel material that is dosimetrically equivalent to tissue and has been used clinically as bolus on skin of patients undergoing radiation therapy. Depth of the jaw was measured to be ∼2.5 cm below the top surface. Duration of irradiation was ∼8 min, dependent on calculated decay of the radiation source. Dose uniformity, especially in the beam direction, was estimated to be roughly 3%.

### Surgery

Both the experimental group and the control group underwent a surgical procedure creating a critical size mandibular defect that has been previously described,^17,20^ with the experimental group undergoing the surgical procedure 4 weeks after the completion of irradiation. Briefly, a midline incision from the mentum to the angles on the mandible was made. Muscle, fascia, and periosteum were reflected to allow for visualization of the mandible. A 10 mm circular trephine bur was used to remove the buccal cortical plate, roots of associated premolar/molar teeth, and the lingual cortical plate, resulting in a 10 mm diameter full thickness cylindrical defect in the left mandible (Fig. 2C). A 1 mm cross cut bur was then used to drill straight through an overlying tooth crown to create an intraoral communication. All drilling was performed under constant irrigation with sterile saline. Muscle and fascia were closed with suture, and rabbits received transdermal Fentanyl patches (25 mcg per hour) and subcutaneous injections of meloxicam (0.3 mg/kg every 24 h) for postoperative pain management (Table 1).

**Table 1.** Preoperative and Postoperative Analgesic Regimens

*Preoperative*	*Postoperative*
Transdermal fentanyl patches (25 mcg per hour)- applied the night before surgery	Subcutaneous meloxicam (0.3 mg/kg every 24 h)- as needed for pain
	Transdermal fentanyl patches (25 mcg per hour)- additional patches as needed for pain
	Subcutaneous buprenorphine (0.02–0.05 mg/kg every 8–12 h)- as needed for pain

### Tissue collection and macroscopic analysis

Four weeks after the surgical procedure was performed, rabbits were euthanized by the intravenous administration of 1 mL Beuthanasia-D^®^ (390 mg/mL pentobarbital solution and 50 mg/mL phenytoin sodium). The left hemimandible and surrounding tissues were harvested and examined for bone stability, degree of soft tissue healing particularly across the site of intraoral communication, and any abnormalities such as abscesses. An ∼2 cm segment of the left mandible, including the 10 mm defect site and ∼5 mm rostral and 5 mm distal to the defect site, was placed in 10% neutral buffered formalin for 48–72 h, rinsed thoroughly with Milli-Q^®^ Ultrapure Water (MilliporeSigma, Burlington, MA), and placed in 70% ethanol solution.

### Microcomputed tomography imaging and analysis

The mandibular bone defects were imaged using a Scanco Medical microcomputed tomography (μCT) 40 micro-CT imaging system (SCANCO Medical, Brüttisellen, Switzerland). Multiple specimens were fixed horizontally in a 36 mm cylindrical sample holder and placed in the *μ*CT specimen chamber. The scanner was set to a resolution of 18 μm/pixel.

The serial tomograms for each specimen were analyzed using TriBON software (RATOC, Tokyo, Japan). Serial tomograms were reformatted in a sagittal orientation to allow for the creation of a standard cylindrical (10 mm diameter × 6 mm depth) volume of interest (VOI) corresponding with the original cylindrical defect created by the trephine bur. Thresholds were set to determine mineralized material in the defect, while excluding background and more mineralized material such as teeth. The volume of mineralized material in the VOI was calculated. Presence or absence of mandibular fracture in the defect area was also determined using *μ*CT analysis.

### Maximum intensity projection scoring

**FIG. 3.**
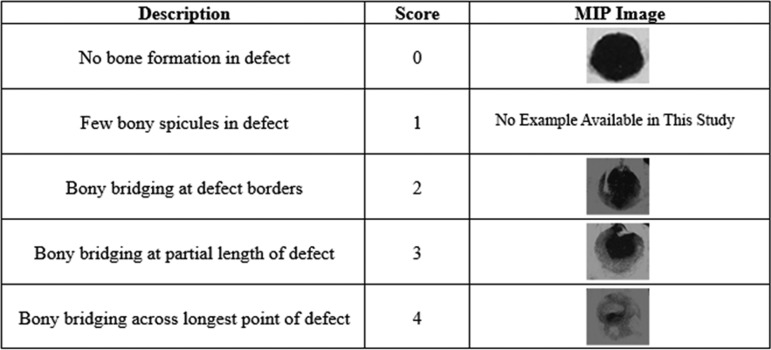
Scoring criteria for bone growth within the CSD. A MIP was scored for each animal as follows: 0, no bone formation in defect; 1, few bony spicules present in the defect; 2, bony bridging present at defect borders; 3, bony bridging at a partial length of the defect; and 4, bony bridging present across the longest point of the defect. MIP, maximum intensity projection.

Maximum intensity projections (MIPs) for each sample were created from the *μ*CT generated DICOM files using OsiriX DICOM Viewer software (Pixmeo SARL, Bernex, Switzerland). Three blinded observers separately graded the MIPs according to a previously published grading scale for the extent of bony bridging^21,22^ and reached a consensus score for each sample. The scale ranges from 0 to 4, with 0 indicating no bone formation in the defect and 4 reflecting boney bridging across the widest point of the defect (Fig. 3).

### Histopathology

**FIG. 4.**
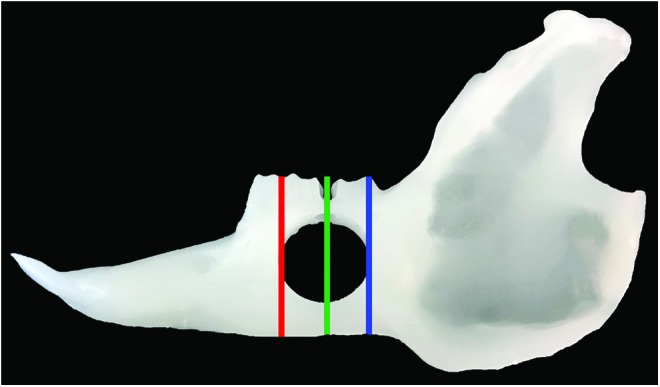
Sectioning for histopathologic analysis. Sections were taken from the anterior of the defect (*red line*, *front*), *middle* of the defect (*green line*, *middle*), and posterior of the defect (*blue line*, *back*). Color images are available online.

After *μ*CT scanning, samples were decalcified in a 14% EDTA solution^23^ for 4–6 weeks. Samples were then sectioned coronally at the anterior (front) and posterior (back) of the defect margins, as well as the center of the 10 mm surgical defect (middle) (Fig. 4). Samples were paraffin embedded, sectioned, and stained with hematoxylin and eosin (H&E) as per standard protocols.

A trained oral and maxillofacial pathologist was blinded and analyzed anterior, middle, and posterior sections for each sample, noting whether signs of compromised wound healing were observed in any sections of the sample. Histopathologic characteristics of compromised wound healing included necrotic bone with empty osteocyte lacunae, marrow fibrosis with hypocellular and hypovascular marrow spaces, nonviable periosteum, and the presence of microorganisms on the surface.^24^ Sections without the previously listed criteria were designated as having no histopathologic signs of compromised wound healing.

### Statistical analysis

All analyses were performed using R statistical software^25^ for evaluation of the differences between the control rabbits and the irradiated experimental rabbits. Fisher's exact test was performed on the histopathology and fracture occurrence data. MIP scoring results were analyzed with an ordered logistic regression and Chi-square test. A generalized linear model and Chi-square test were used to analyze the *μ*CT bone volume data.

## Results

### Macroscopic characteristics of surgical defect

The irradiation and surgical procedure were well tolerated by all animals, with no significant clinical abnormalities noted during the duration of the study. At the time of tissue collection, samples of the defect were observed for macroscopic abnormalities. Mobility of the bone in the collected sample site was noted in three irradiated animals compared to one control animal. Small abscesses were noted near the subcutaneous suture site in three irradiated animals compared to one control animal. Two of the irradiated animals with noted mobility at the defect site had uneven wear on incisors and molars.

### Quantitative assessment of bone healing in surgically created mandibular defect

**FIG. 5.**
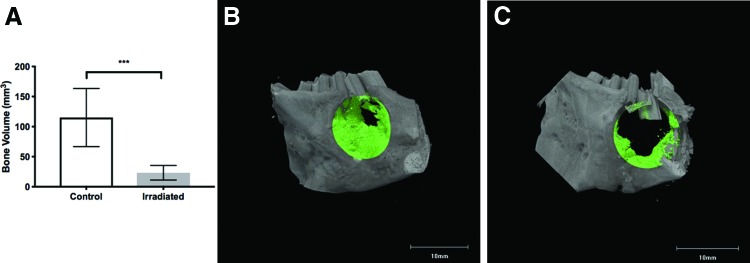
**(A)** Quantitative evaluation of bone volume (mm^3^) in cylindrical bone defect. Chi-square test, ***p ≤ 0.001. *μ*CT generated sagittal tomograms showing representative defect healing. Bone volume highlighted in *green*. **(B)** Control animal defect and bone healing. **(C)** Irradiated animal defect and bone healing. Color images are available online.

The volume of bone in the VOI, representing the surgically created defect, was determined 4 weeks after creation of the defect. The mean bone volume in the control animals was 115.0 ± 15.3 mm^3^ compared to 23.2 ± 3.9 mm^3^ in irradiated animals (Fig. 5). The difference was statistically significant when comparing irradiated animals to unirradiated controls (*p* ≤ 0.001).

*μ*CT was also used to confirm presence of fractures in or around the surgical defect site. While 70% of irradiated animals had mandibular fractures compared to 20% of the control animals, the difference between the groups was not statistically significant (*p* = 0.06).

### Assessment of bone healing with a standardized scoring system

MIPs were created for each sample and scored based on a previously published scoring system, with 0 being no bone formation and 4 being bony bridging over the longest point of the defect.^21,22^ The control group had significantly higher scores than the irradiated group (*p* ≤ 0.001) (Table 2).

**Table 2.** Maximum Intensity Projection Scores

*MIP scores*	*Description*	*Number of control animals*	*Number of irradiated animals*
0	No bone formation in defect	0	1
1	Few bony spicules in defect	0	0
2	Bony bridging at defect borders	2	8
3	Bony bridging at partial defect length	7	1
4	Bony bridging across longest defect point	1	0

MIP, maximum intensity projection.

### Histopathologic characterization of animal model

**FIG. 6.**
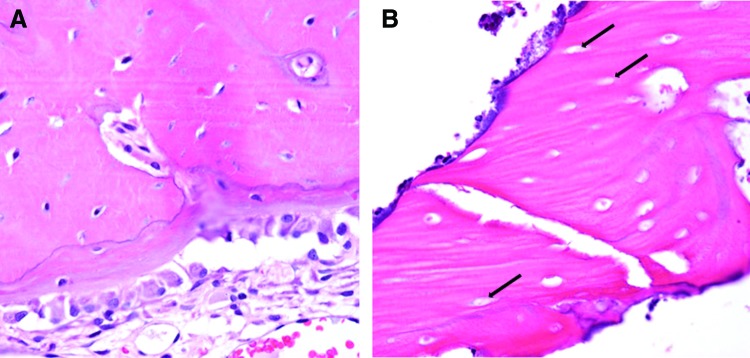
H&E stained photomicrographs showing representative histopathology findings, 40 × . **(A)** Control animal defect showing bone with osteocytes in lacunae and healthy osteoblast lining. **(B)** Irradiated animal showing necrotic bone with empty lacunae (*arrows*). H&E, hematoxylin and eosin. Color images are available online.

H&E stained sections of the defects were analyzed for histopathologic features of compromised healing. All 10 animals of the control group had no signs of comprised wound healing, while eight of the irradiated group did have features indicative of compromised healing (Fig. 6). There was a statistically significant difference in the histopathologic impression between the control and irradiated group (*p* ≤ 0.001).

## Discussion

Maxillofacial injuries often present a unique challenge to clinicians. Large soft tissue and bone loss, combined with an often compromised wound environment, can lead to failures of traditional reconstruction techniques.^1,26^ Tissue engineering technologies offer potential alternatives for improved patient outcomes in these maxillofacial defect scenarios. However, to more accurately predict clinical patient outcomes, the preclinical *in vivo* models must closely mimic the complex comprised wound healing environment often seen in these patients.^10,12^

A variety of preclinical animal models exist that attempt to create a comprised maxillofacial wound healing environment through radiation which have been reported. Rodent radiation and bone healing models often lack a CSD, a maxillofacial environment that accurately mimics movement and stresses experienced by human patients, and involve a less standardized surgical procedure, such as minimally described tooth extraction surgical techniques, that may be influenced by intersurgeon variability and may result in differences in tissue damage between animals.^27–29^ Previously described preclinical rabbit models using radiation to create a compromised wound environment also have similar shortcomings,^16,30,31^ such as a lack of a CSD in the maxillofacial environment or a well-characterized surgical technique. This model utilizes a surgically reproducible technique as previously described, in which a CSD is created with a trephine bur, mitigating variability and enabling consistent defect creation between animals.^17,20^ It was hypothesized that when combined with radiation, the surgical technique would allow for creation of a consistent CSD in a compromised maxillofacial wound environment.

While some animals experienced fractures at the surgical sites, no clinical abnormalities were noted for the duration of the study and procedures were well-tolerated by all animals. Whether the fractures occurred antemortem or were a result of tissue collection and manipulation of weakened bone is difficult to determine. While no sign of callous formation or fracture healing was noted on *μ*CT or other analyses, which would suggest a more chronic fracture, radiation can affect proper callous formation and bone healing.^32,33^ Subsequent studies may benefit from postoperative radiographs to assess margin integrity radiographically or fixation hardware, which has been previously utilized.^20^

*μ*CT results showed significantly decreased bone volume in the defect site in irradiated animals, confirming comprised healing in the bone defect site. Altered wound healing in human postradiation treatment is well-documented, with healing of skin and other tissues influenced by radiation effects on vasculature, fibroblasts, and growth factors.^34,35^ Studies in animals show similar results, with radiation being shown to impact multiple components of the healing process, such as osteogenesis and angiogenesis.^16,29,30,36^ However, the exact pathogenesis of radiation injury leading to impaired wound healing is not fully understood and is likely influenced by a variety of substances such as cytokines and growth factors.^37^ Future research looks to further characterize this animal model through investigation of differences in growth factors between control and irradiated animals.

Histopathologic analysis showed hallmarks of bone necrosis in irradiated animals, such as lacunae void of osteocytes and a lack of osteoblastic lining, which may have led to overall weakened bone structure.^38^ Histopathologic findings indicate a possible underlying necrotic process leading to compromised wound healing. Evidence of necrosis in this animal model is suggestive of the human clinical condition of osteoradionecrosis, a debilitating complication of nonhealing wounds that can affect patients that undergo oral manipulations after previous radiation treatments.^39–41^ Forthcoming studies look to characterize this animal model as a potential preclinical model of osteoradionecrosis.

Osteonecrosis of the mandible can occur not only secondary to radiation but also to a variety of inciting stimuli. Bisphosphonates, osteoclast inhibitors used to treat osteoporosis, have previously been associated with cases of osteonecrosis of the jaw.^42,43^ Other ailments, such as diabetes mellitus, have been shown to be associated with medication-related osteonecrosis of the jaw.^44^ A variety of animals have been developed to mimic these forms of osteonecrosis in an *in vivo* translational setting.^45,46^ Due to the well-documented histopathologic differences and varying methods of development,^24^ it is unclear how accurately this presently described model mimics nonradiation forms of mandibular osteonecrosis.

Translation of results seen in *in vivo* research to human clinical trials remains a major challenge, with the majority of human studies failing to replicate results.^47^ The inability of animal models to adequately mimic clinical scenarios and the difficulty of reproducing experimental protocols are frequently highlighted, particularly in radiation-related research.^48,49^ This study combined radiation with a critical size mandibular defect to create a consistent model of comprised oral wound healing in rabbits. Utilization of this animal model that more accurately mimics human clinical patients who experience compromised wound healing secondary to radiation may lead to better clinical success of tissue engineering techniques with promising *in vivo* results. Future research avenues include utilizing the model to determine efficacy of novel tissue engineering techniques in the comprised oral environment.

In conclusion, this animal model combining a radiation-induced comprised maxillofacial healing environment with a previously determined CSD has a potential for improved translational research of tissue engineering technologies *in vivo*.
